# Effects of Thermal Manipulation and Serotonin Modulation on Brain HSP70 and HSP90 Gene Expression in Late Embryogenesis of Broilers

**DOI:** 10.1002/vms3.70195

**Published:** 2025-04-02

**Authors:** Hamed KHasti, Ladan Emadi, Shahrzad Azizi, Elham Mohammadi, Hadi Tavakkoli

**Affiliations:** ^1^ Department of Basic Sciences, Faculty of Veterinary Medicine Shahid Bahonar University of Kerman Kerman Iran; ^2^ Department of Basic Sciences, Faculty of Veterinary Medicine University of Tehran Tehran Iran; ^3^ Department of Pathobiology, Faculty of Veterinary Medicine Shahid Bahonar University of Kerman Kerman Iran; ^4^ Department of Clinical Sciences, Faculty of Veterinary Medicine Shahid Bahonar University of Kerman Kerman Iran

**Keywords:** brain, chicken embryo, heat stress, HSPs, serotonin

## Abstract

**Introduction:**

Broiler chickens are particularly vulnerable to elevated temperatures compared to mammals because they have feathers instead of sweat glands, undergo rapid growth and are intensively bred in commercial systems. Serotonin, as neurotransmitter, is essential for the development of the embryonic brain and neural functions, helping the body adapt to difficult environments such as heat stress (HS) that broiler chickens are susceptible to by regulating physiological and metabolic processes. Heat shock proteins, which are produced in response to different types of stress, protect cells from damage. This research seeks to investigate the effect of HS on the cellular stress response in embryonic brain tissues, with a particular emphasis on the role of serotonin.

**Methods:**

A total of 120 fertilized eggs were randomly divided into control and serotonin (20 µg/egg) groups. Before incubation, serotonin solution or normal saline (0.9% NaCl) was injected into the albumen. On the 13th day of the experiment, subjects were divided into groups exposed to either high or normal temperature conditions. The HS groups were initially exposed to 39.5°C for 2 h, with the exposure duration increasing by 2 h each day until the 17th day of incubation, culminating in 10 h of HS on the final day. On the 18th day, brain samples were collected for histopathological examination and mRNA expression analysis of HSP70 and HSP90.

**Results:**

HS significantly reduced the gene expression of HSP70 and HSP90 in embryonic brain tissue. However, the presence of serotonin under stress conditions significantly increased the expression of these heat shock proteins compared to the HS group alone.

**Conclusion:**

This study is the first to report decreased gene expression of brain HSP70 and HSP90 in Ross broiler embryos under HS, with serotonin serving as an anti‐stress agent by promoting HSP gene expression. Further research is necessary to explore the effects of serotonin on heat tolerance and chick performance post‐hatching.

## Graphical abstract

This study is the first to report decreased gene expression of brain HSP70 and HSP90 in late embryogenesis of Ross broiler under heat stress, with serotonin serving as an anti‐stress agent by promoting HSP gene expression.

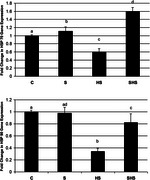

## 1 Introduction

The demand for poultry meat is projected to rise in the coming decades as the global human population continues to grow (OECD/FAO 2023). To ensure sustainable high production efficiency within the supply chain, hatcheries must focus on maximizing the hatchability of healthy chicks. Temperature, as an important environmental condition that impacts several biological functions and behavioural activities of birds, is considered the most critical incubation condition (Han et al. 2022; Rocha et al. 2022; Yalcin, Özkan, and Shah 2022). Heat stress (HS) increases body temperature and induces oxidative stress (Chowdhury et al. 2014; Yang et al. 2016) which affects metabolism and the function of various organs in broilers (Wu et al. 2016). Thermal manipulation (TM) during chicken egg incubation has been demonstrated to significantly improve birds’ metabolism, post‐hatch thermoregulation and reduce the adverse effect of high ambient temperature (Han et al. 2022; Ramiah et al. 2022). Thermally manipulated fertile eggs during incubation (a critical developmental period for embryos), especially from the 12th to the 18th day, have been shown to improve growth performance and reduce the negative impacts of post‐hatch HS by enhancing thermotolerance acquisition (Al Amaz et al. 2024; Iraqi et al. 2024). Additionally, early heat acclimation, whether occurring during prenatal or postnatal stages, can epigenetically modulate gene expression, resulting in a long‐term physiological memory that improves thermotolerance (Al Amaz et al. 2024; Nichelmann and Tzschentke 2002; Ramiah et al. 2022). The formation and maturation of the hypothalamic–hypophyseal–adrenal axis (ED 10–16) (M.‐B. Al‐Zghoul, Abd Elhafeed, et al. 2015; Piestun et al. 2008) and the hypothalamic–hypophyseal–thyroid axis (ED 7–16) (M.‐B. Al‐Zghoul, Abd Elhafeed, et al. 2015; Piestun et al. 2011) in chickens play a crucial role in regulating thermoregulation and stress responses. Consequently, TM during late‐term embryogenesis may activate stress response mechanisms in broilers. Embryos make some temperature adjustments during development, but their thermoregulatory system is not fully formed. Therefore, environmental temperature affects embryonic development, hatchability, chick quality and later bird performance (Faria Filho et al. 2008).

Researchers and producers need to persist in exploring optimal incubation conditions tailored to their specific strains, while hatcheries must enhance their efficiency to meet the demands of contemporary chick genotypes. The temperature requirements for embryo incubation vary throughout embryogenesis, depending on the strain and age (M. B. Al‐Zghoul et al. 2023; Rocha et al. 2022; Tona et al. 2022). In recent years, research has focused on the effects of TM during incubation on thermoregulatory mechanisms and the expression of genes involved in the response to thermal stress, such as those encoding heat shock proteins (HSPs) or stress proteins. HSPs are a group of highly conserved proteins produced in response to physical, chemical, or biological stresses, including heat exposure. They organize the heat shock response (HSR), acting as a regulatory pathway to protect cells from various stressors (Wu et al. 2016). HSPs are a diverse class of molecular chaperones with various molecular weights and biological roles. Small HSPs (molecular weight 540 kDa) and the HSP60, HSP70, HSP90 and HSP100 protein families are typical divisions of HSPs. These families all operate as chaperones, facilitating the folding, unfolding and refolding of newly formed or stressed‐out proteins to stop erroneous folding and additional deterioration and damage (Belhadj Slimen et al. 2014). Research suggests that the degree of thermal tolerance is associated with the expression level of HSPs (Krebs and Bettencourt 1999). TM has been shown to mitigate the adverse effects of post‐hatch HS by improving thermotolerance acquisition during embryogenesis (Al Amaz et al. 2024). Higher thermotolerance can be achieved by rapid thermal stress response, acclimation and epigenetic temperature adaptation (Yahav 2009).

Serotonin (5‐hydroxytryptamine, 5‐HT) plays a role in the stress response across all animals, and elevated serotonin release regulates physiological and metabolic adjustments to challenging conditions (Chaouloff, Berton, and Mormède 1999; Joëls and Baram 2009). Research indicates that serotonergic signalling is crucial for the expression of HSP70 mRNA after heat shock, suggesting it may function as a signalling mechanism to activate the HSR. Certain studies have noted that HS leads to a decrease in serotonin levels in regions such as the hypothalamus, midbrain and brain stem (Ahmed‐Farid et al. 2021; Calefi et al. 2019). Additionally, acute stressors, like environmental temperature increases, have been reported to elevate serotonin concentrations in brain tissues. However, under chronic stress conditions, the level of brain serotonin tends to decrease (Calefi et al. 2019).

Given the established effects of HS on the expression of HSP genes and the role of serotonin in stress modulation and HSP expression, the present study aimed to explore the impact of TM and in ovo serotonin on the expression of HSP70 and HSP90 genes in the brain during late‐term embryogenesis.

## 2 Materials and Methods

### 2.1 Experimental Design and Sample Preparation

A total of 120 fertilized eggs (Ross 308; weighing 55 ± 2 g) were obtained from the Mahan Poultry Center in Kerman and placed in an incubator (Cocks Machine Co. Ltd., Iran) with automatic rotation every 2 h during the entire incubation period and maintaining a constant incubation temperature of 37.5°C and a relative humidity of 55%–65%. The eggs were divided into two groups (60 eggs/group): control (C), which received 50 µL of normal saline (0.9% NaCl) (Darou Pakhsh, Pharmaceutical Mfg. Co. Tehran, Iran) per egg, and serotonin (S), which received 20 µg of serotonin in 50 µL of normal saline per egg. The injection of either serotonin solution or normal saline into the albumen was carried out before incubation (Dennis, Fahey, and Cheng 2013; Huang et al. 2021). After disinfecting and creating a small hole at the larger end of each egg, sterile normal saline or serotonin solution was injected to a depth of 25 mm using a 1‐mL disposable syringe with a 25‐gauge needle. Following the injection, the small holes were promptly sealed with Scotch tape, and the eggs were returned to the incubator for further incubation. Unfertilized eggs were identified through candling and discarded on the 7th day of incubation (Han et al. 2019).

On the 13th day of the incubation, each group was assigned to either stress or normal temperature conditions. The HS groups were exposed to stress conditions at 39.5°C for 2 h initially, with the duration of stress increasing by 2 h daily until the 17th day of incubation (10 h of HS on the 17th day). These specific days were chosen based on the establishment of the functional pituitary–adrenal axis and the initiation of development in the hypothalamohypophyseal–thyroid axis (Moraes et al. 2003). The serotonin–heat‐stressed group (SHS) received serotonin prior to incubation, and HS condition was induced from the 13th day according to the HS group.

On the 18th day, the embryos were euthanized under anaesthesia using isoflurane, following which embryonic brain samples were collected. These samples were promptly frozen in liquid nitrogen and stored at −80°C for further analysis (Han et al. 2019). All procedures were performed in compliance with the Guide for the Care and Use of Laboratory Animals by the National Academy of Sciences (National Institutes of Health Publication No. 86‐23).

### 2.2 RNA Isolation and cDNA Synthesis

The embryonic brain tissue's total RNA was extracted using the Total RNA Extraction Kit (Parstous Co. Ltd., Iran) according to the manufacturer's instructions. The integrity and purity of the RNA samples were evaluated using agarose gel electrophoresis and nanodrop spectrophotometry (Eppendorf Company, Germany), respectively. Following this, reverse transcription was performed using the Easy cDNA Synthesis Kit (Parstous Co. Ltd., Iran), following the manufacturer's protocol and utilizing 1 µg of total RNA.

### 2.3 Evaluation of HSP70 and HSP90 mRNA Expression Level

To evaluate the expression levels of HSP70 and HSP90 in the broiler embryo brain, real‐time PCR (qPCR) was performed using the 2X SYBR Green Real‐Time PCR Kit (Parstous Co. Ltd., Iran) on a LightCycler Detection System (Roche Company, Germany). The relative expression levels of the target genes were normalized to chicken GAPDH, which served as a housekeeping gene. The primer sequences used for qPCR are detailed in Table 1 (Han et al. 2019; Rajkumar et al. 2015). Reactions were carried out in a 20 µL mixture comprising 10 µL of SYBR Green Real Time PCR (2X), 0.4 µM of each primer (0.8 µL), 1 µL of cDNA and 7.4 µL of nuclease‐free water. The cycling conditions included an initial denaturation at 95°C for 10 min, followed by 40 cycles of denaturation at 95°C for 30 s, annealing at 57°C for 30 s and extension at 72°C for 20 s. All reactions were performed in triplicate. Additionally, the melting stage was executed as follows: 95°C for 10 s, 65°C for 60 s and 97°C for 1 s. Relative quantification was conducted utilizing the comparative 2‐ΔΔCt method.

**TABLE 1:** Sequence of primers used for real‐time PCR.

Gene	Sequence 5′–3′ (forward/reverse)
HSP70	5′‐GGGAGGACTTTGACAACCGA‐3′/5′‐CAAAGCGTGCACGAGTGATG‐3′
HSP90	5′‐GAAGACTCCCAGAACCGCAA‐3′/5′‐ACCTGGTCCTTTGTCTCACC‐3′
GAPDH	5′‐CTGCCGTCCTCTCTGGC‐3′/5′‐GACAGTGCCCTTGAAGTGT‐3′

### 2.4 Histopathological Evaluations

Five sections, each with a thickness of 5 µm, were prepared from each paraffin‐embedded sample. These sections were then stained using the haematoxylin and eosin (H&E) method and then evaluated under light microscopy (BX50, Olympus, Japan) (Xu et al. 2018).

### 2.5 Statistical Analysis

The results of HSPs expression were analyzed by using the GLM procedure of SPSS 16.0 (SPSS Inc., Chicago, IL, USA) package. Evaluation of significant difference between the means of different experimental groups was performed using one‐way analysis of variance (one‐way ANOVA) followed by the Tukey's test as post hoc. Values were expressed as mean ± SEM. The significance considered level was *p* < 0.05. The histopathologic observations were subjectively evaluated between the groups.

## 3 Results

### 3.1 HSP70 and HSP90 Gene Expression

In the HS condition, a significant decrease in embryonic brain HSP90 and HSP70 mRNA expression was observed compared to the control group. However, in ovo injection of serotonin significantly increased mRNA expression levels of both HSP70 and HSP90 compared to the control temperature group, particularly under HS conditions.

**FIGURE 1:**
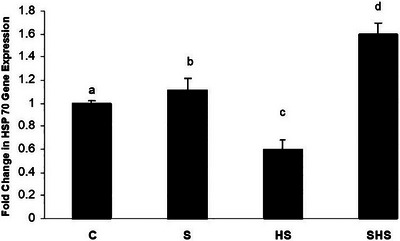
The expression level of HSP70 relative to GAPDH in the broiler embryo brain. Groups with different superscript letters denote significant differences at *p* < 0.05. C: normal control, HS: heat stress, S: serotonin, SHS: serotonin and heat stress.

**FIGURE 2:**
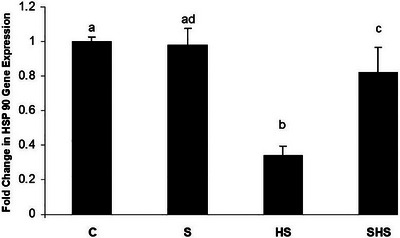
The expression level of HSP90 relative to GAPDH in the broiler embryo brain. Groups with different superscript letters denote significant differences at *p* < 0.05. C: normal control, HS: heat stress, S: serotonin, SHS: serotonin and heat stress.

The results of mRNA expression levels of HSP70 and HSP90 are shown in Figures 1 and 2.

### 3.2 Brain Histopathology

**FIGURE 3:**
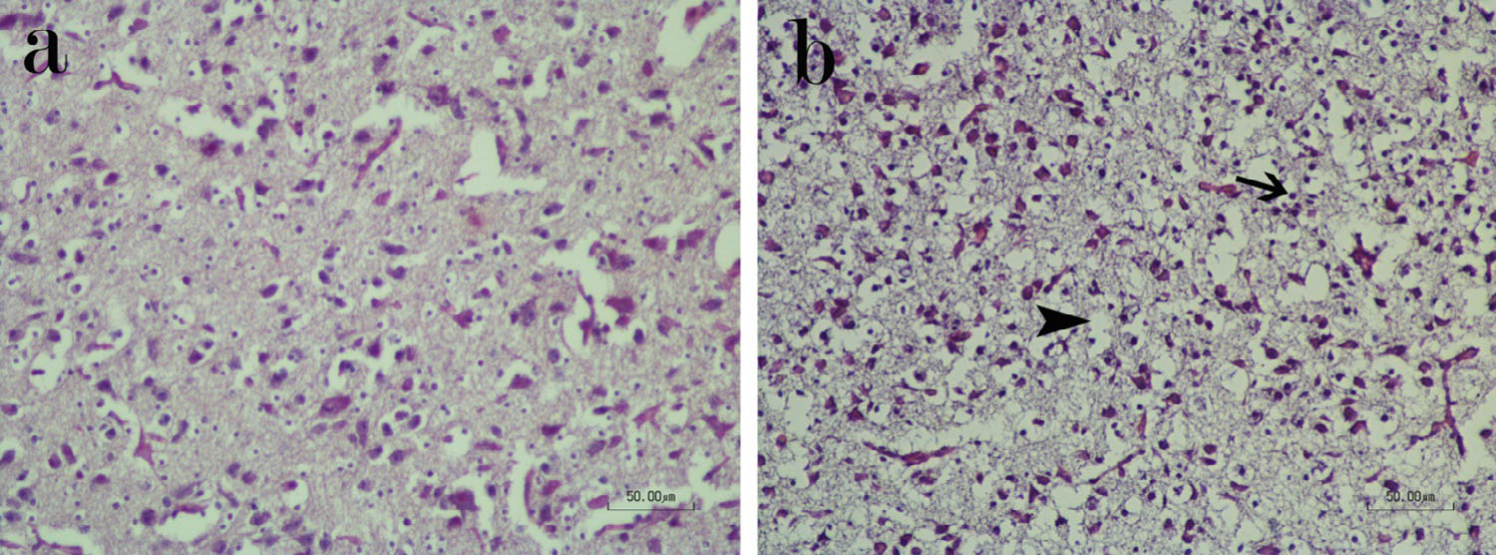
Brain sections of chicken embryo. (a) Normal control group. (b) Heat stress group shows increasing glial cells (arrow) and status spongiosus appearance that is characterized with hollow spaces (arrowhead) (HE staining, scale bar = 50 µm).

In the control group (C group), the embryonic sections of the cerebrum on the 18th day of incubation exhibited round to oval neurone cell bodies with a normal histological appearance. Neurone nuclei generally appeared euchromatic with prominent nucleoli. Pyramidal neurones displayed a more basophilic nucleus and eosinophilic cytoplasm compared to other neurones. The boundaries between different layers were not distinctly separate. Glial cells were distributed throughout the brain. Additionally, some cells with dark eosinophilic cytoplasm and heterochromatic nuclei were observed in the brain (Figure 3a). In the normal temperature group receiving in ovo injection of serotonin (S group), the histology of the embryo brain was similar to that of the normal control group, showing no pathological changes.

Brain sections of embryos in the HS (HS group) exhibited higher cellularity compared to the control group, primarily due to an increase in glial cells. The glial cells appeared round and displayed heterochromatin. The increase in glial cells was observed diffusely throughout the brain, as well as in some focal areas. Additionally, a status spongiosus change was noted in various areas of the brain, characterized by scattered hollow spaces. Oedema was another lesion observed, affecting the brain tissue (Figure 3b).

**FIGURE 4:**
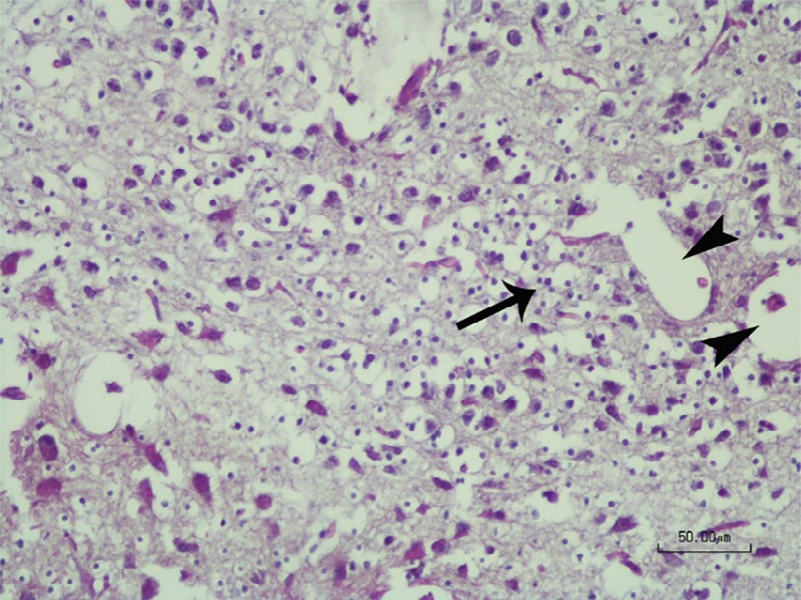
Brain of chicken embryo exposed to in ovo serotonin injection shows gliosis (arrow) and dilation of Virchow–Robin space (arrowheads) due to oedema in heat stress condition (HE staining, scale bar = 50 µm).

In the HS condition, the in ovo injection of serotonin (SHS group) did not exhibit any apparent advantage or disadvantage in terms of histopathological lesions. The pathological changes observed in this group were similar to those in the HS group, characterized by gliosis, dilation of Virchow–Robin space due to cerebral oedema and status spongiosus (Figure 4).

## 4 Discussion

HS poses a significant challenge to domestic animal production, especially in commercial poultry (Cedraz et al. 2017). Additionally, among the HSP families, HSP70 and HSP90 have been extensively studied, with their gene expression serving as a biomarker of thermal stress or oxidative stress in organisms (El Golli‐Bennour and Bacha 2011; Tedeschi et al. 2015). Therefore, the present study aimed to investigate the effects of HS (39.5°C) during embryogenesis from the 13th to the 17th day for 5 days (starting with 2 h on the 1st day and increasing up to 10 h on the 5th day), as well as the modulation of HSP70 and HSP90 gene expression by in ovo serotonin.

The findings of the current study revealed decrease in the expression of HSP70 and HSP90 genes as a result of TM during embryogenesis. However, in ovo serotonin injection led to induction of HSP70 and HSP90 gene expression on Egg Day 18. Histopathological examination of the brain demonstrated neuronal necrosis, gliosis, status spongiosus and oedema in all groups under HS conditions.

TM during egg incubation is recognized as a form of mild heat shock exposure during embryogenesis, which enhances tissue stability, oxidative stress response and immune responses to HS (M. B. Al‐Zghoul et al. 2023). Various TM protocols have led to a significant increase in the expression of HSPs mRNA in both pectoral and thigh muscles (M.‐B. Al‐Zghoul, Ismail, et al. 2015; M.‐B. Al‐Zghoul et al. 2013; Ali et al. 2022; Ramiah et al. 2022). This increase has been linked to improved thermoregulation and thermotolerance, as noted by Al‐Aqil and Zulkifli (2009). Understanding how TM affects the chicken embryo's brain and its relationship with HSP induction in the development of thermotolerance is essential. The HSR involves heightened production of cytoprotective HSP70 and decreased cytokine levels with repeated heat exposure, resulting in reduced markers of cellular and systemic heat strain (Kuennen et al. 2011; Ramiah et al. 2022).

Thermal adaptation during embryogenesis to high temperatures has been considered one of the strategies to mitigate HS during postnatal life, aiming to enhance heat tolerance, particularly in broiler chicks (Rajkumar et al. 2011). The activation of the hypothalamic–pituitary–adrenal (HPA) axis in the chicken embryo is thought to occur during the mid‐ or late‐embryonic period. At this stage, raising the incubation temperature could cause associated genes to undergo epigenetic changes that alter the threshold for the hypothalamus temperature set point, changing chickens’ response to HS (Dhahir 2016).

It was demonstrated that embryonic brain small HSPs, including HSPB1, HSPB5 and HSPB8, were downregulated in response to heat (41°C) or cold (33°C) stress conditions from the 15th to the 17th day, with 3 h of stress exposure each day during incubation. However, HSPB9 was upregulated in the HS group. Mild degeneration in the cerebrum was observed upon histopathological evaluation (Basaki et al. 2020). It has been noted that changes in the expression of HSP genes in thermally manipulated birds might be linked to alterations in methylation at different sites within the promoter regions of these genes. Typically, HSPs are upregulated in response to stress and return to baseline levels once the stressors are eliminated. The differences observed in the timing of stress response regulation could be influenced by factors like species, type of stressor, tissue type and the specific HSP under investigation (Mymrikov, Seit‐Nebi, and Gusev 2011; Sugiyama et al. 2000).

The impacts of prolonged HS on diverse physiological and immunological parameters across different breeds of broilers were evaluated. It was observed that elevated temperatures led to mild inflammatory cellular hyperplasia in the brain, leg muscles and heart. Furthermore, the levels of HSP70 transcripts were heightened in broilers exposed to HS. These results suggested an augmentation in the expression of HSP70 mRNA in broilers experiencing HS conditions (Xu et al. 2018). On the other hand, the expression of HSPs can vary depending on embryonic age. Studies have indicated that the highest expression of HSP90 was observed on Egg Days 14 and 18, while HSP60 showed peak expression on Egg Days 14 and 16 in the brain (M.‐B. Al‐Zghoul, Ismail, et al. 2015).

The cellular mechanisms' response to the long‐term effects of thermal conditioning is complex, and conflicting observations have been reported in earlier studies. One perspective suggests that elevated HSP70 expression is associated with improved heat tolerance (Wang and Edens 1993; Wang and Edens et al. 1998), while an opposing viewpoint links it to a low level of expression (Yahav, Shamay, et al. 1997).

The lower expression of HSP in heat‐exposed birds suggests the potential impact of thermal adaptation, particularly since the chicks were exposed to higher incubation temperatures during the embryonic stage (Rajkumar et al. 2010). This is supported by findings demonstrating reduced HSP70 gene expression in heat‐exposed birds compared to normal birds (Piestun et al. 2008; Yahav 2009).

Based on the low expression of HSP observed in the present study following 5 days of HS during embryogenesis, it's plausible to hypothesize that thermal adaptation and heat tolerance may indeed improve even during the embryonic period. This suggests that exposure to elevated temperatures during embryogenesis could potentially induce adaptations that enhance the organism's ability to cope with HS later in life.

It's recognized that the thermoregulatory system of birds begins to develop during the first 18 h of incubation, concurrent with the formation of the central and peripheral nervous systems. This system is responsible for thermoregulatory mechanisms and initiates as soon as the neural canal completely closes (around 48 h) (Faria Filho et al. 2008).

Previous research has demonstrated significant increases in protein expression levels of HSP90, HSP60 and HSP70 under chronic HS conditions (15–42 days, 35°C) (Vinoth et al. 2015) with HSP70 also being upregulated in acute HS conditions in broilers (30 days, 36°C, 3 h) (Soleimani et al. 2011). These findings suggest that HS may indeed elevate HSP expression in broilers. However, conflicting effects of early heat treatment on HSP expression have been reported in other studies. For instance, no significant effect of initial heat exposure on HSP70 expression was observed from 1 to 21 days (36°C, 1 h/day) (Yahav, Shamai, et al. 1997). Additionally, some studies have reported no effects of initial heat treatment (5‐day‐old, 36°C, 24 h) on the expression of HSP70 or HSP90 when HS was applied just before marketing (Toplu, Nazligül, et al. 2014). Similarly, lower HSP70 expression has been noted in the liver, brain and kidneys of early heat‐treated (5‐day‐old, 36°C, 24 h) chicks following chronic HS (22–42 days, 35°C, 6 h/day) compared to the group subjected to chronic HS without early heat treatment (Toplu, Tunca, et al. 2014). These inconsistent results may be attributed to variations in broiler breeds, sample collection methods, heat treatments and environmental conditions.

HSPs are associated with corticosterone levels and growth performance. Elevated levels of HSP70 are observed under stressful conditions and serve as an indicator of HS. HSP70 protects cells by preventing protein function degradation through the inhibition of protein aggregation. However, the synthesis of HSPs comes at a physiological cost, as it diminishes the availability of other proteins, thereby negatively affecting growth (Mahmoud and Edens 2005).

In mammals, placental serotonin serves as the primary source of 5‐HT for foetal brain development during early embryogenesis (Bonnin, Goeden, et al. 2011). The foetal 5‐HT level is influenced by maternal metabolism, primarily through maternal circulating tryptophan, the precursor of 5‐HT in serotoninergic neurones. This regulation is crucial for participating in synaptogenesis and neuronal maturation during mid–late gestation (Bonnin and Levitt 2011). Chicken embryos exhibit a developmental pattern in 5‐HT similar to that of humans and rodents (Hynes and Rosenthal 1999). However, they are shielded from background influences such as maternal metabolism effects during embryogenesis and are easily accessible (Hynes and Rosenthal 1999). Therefore, the chicken embryo provides an ideal model for investigating the mechanisms of prenatal 5‐HT exposure and its related long‐lasting effects on physiological homeostasis and behavioural outcomes in both humans and animals.

Moreover, experimentally manipulating serotonin signalling in mammalian models can result in heightened production of stress hormones and trigger aversive behaviours, even in the absence of an actual stressor (Chaouloff, Berton, and Mormède 1999; Joëls and Baram 2009). Thus, while it remains uncertain whether increased serotonin release is specific to certain stressors or a nonspecific response associated with heightened arousal, serotonergic signalling allows organisms to rapidly adjust their physiology, metabolism and behaviour in anticipation of imminent danger. Exposure to adverse environments can also lead to macromolecular damage (Bukau 1993; Somero 1995).

Thyroid hormones play a significant role in the adaptation to HS by regulating the metabolic rate of birds during growth and egg production (M.‐B. Al‐Zghoul, Ismail, et al. 2015). Serotonin may exert its effects by interfering with the release of other hormones, such as thyroid hormones, during embryogenesis (McNabb and King 1993; Renden et al. 1994). This suggests a potential mechanism through which serotonin influences physiological responses to HS, possibly by modulating the release or activity of thyroid hormones.

In prior studies, TM resulted in a notable elevation in mRNA levels of HSP90, HSP60 and HSF‐1 in muscle, heart and brain tissues of chicks during embryogenesis (M.‐B. Al‐Zghoul, Ismail, et al. 2015). Furthermore, acute exposure to high ambient temperatures increased the mRNA expression of HSP70 and HSP90 in the chicken embryo brain (Yang et al. 2016). These findings underscore the significance of HSPs in responding to thermal stress during embryonic development. Indeed, it has been reported that on post‐hatch Day 1, the mRNA levels of HSP70 were significantly lower in TM compared to control conditions (M. B. Al‐Zghoul 2018). This suggests that the expression of HSP genes may be age‐dependent and lower in embryos and neonatal chicks. This variation in expression levels across different developmental stages underscores the dynamic nature of the HS response and the complexity of the regulatory mechanisms involved. Previous research has shown that TM exerts both short‐ and long‐term effects on gene expression, as evidenced by elevated basal HSP70 expression in the TM groups compared to the control group (M. B. Al‐Zghoul 2018). This suggests that TM can induce enduring alterations in the expression of HSPs, potentially providing increased tolerance to thermal stress in the manipulated groups.

Incubating broiler embryos at 40°C for 4–6 h on the 13th, 16th and 19th days of incubation led to HSP70 expression two to five times higher in the brain compared to other embryonic tissues. Additionally, younger embryos (on Day 13 of incubation) exhibited higher HSP70 synthesis than older embryos. HS also resulted in increased HSP70 expression in the heart of embryos on the 13th and 19th days of incubation. These results suggest that HSP70 protein expression in broiler embryos varies depending on tissue, embryonic age and thermal stimulation (Leandro et al. 2004). In another study, the expression of HSP90 alpha, HSP90 beta and HSP60 mRNA proteins was examined in broiler chickens subjected to incubator temperatures of 39.5°C for 3 h on the 16th, 17th and 18th days of incubation. The study found that the expression of HSP genes in response to thermal stimuli varied depending on the type of HSP, tissue examined, breed and age of the birds. The authors concluded that decreased tissue HSP levels may contribute to increased heat tolerance in broilers (Rajkumar et al. 2017).

TM during embryogenesis (prenatal) induces physiological memory through epigenetic adaptation to high temperatures, resulting in improved thermotolerance during postnatal life (Yahav 2009). Additionally, cyclical higher incubation temperatures have been shown to enhance heat tolerance in chickens, with the effectiveness depending on the duration and timing of exposure (Nadeau and Landry 2007). These findings underscore the importance of early‐life thermal experiences in shaping the thermotolerance of avian species.

Supplementation of nutrients, including vitamins and minerals, has been demonstrated to effectively regulate the expression of HSP70 in animals. Various antioxidants have been utilized to mitigate the effects of HS on animals. For instance, supplementing selenium in turkey embryos minimized the consequences of HS by maintaining hepatic HSP70 expression, which would otherwise be heightened when birds were exposed to 40°C for 2 h (Rivera et al. 2005). Similarly, organic selenium (selenium yeast) fed to broilers challenged with enteropathogenic *Escherichia coli* resulted in reduced hepatic HSP70 expression under HS at 40°C for 1 h, illustrating selenium's ability to mitigate HS responses even under challenging conditions (Mahmoud and Edens 2005). Additionally, dietary vitamin C supplementation has been found to decrease the expression of HSP70 in heat‐stressed chicks. The role of antioxidants in modulating the expression of proinflammatory and antioxidant genes has already been established (Costa et al. 2020). Therefore, the use of supplementary nutrients is recommended for better management of stress in animals.

## 5 Conclusion

This study represents the first documentation of decreased brain HSP70 and HSP90 gene expression in Ross broiler embryos under HS condition. This reduction may be attributed to heat adaptation during the TM period and method employed, or the development of other thermoregulatory mechanisms such as thinner skin and increased vascularity, resulting in fewer signs of HS. Also, it's possible that the Ross strain exhibits thermotolerance compared to other breeds. In addition, effective induction of HSP expression by serotonin can improve thermal tolerance through cell protection during the embryonic period or after hatching in different HS scenarios. Investigation on the effects of different TM techniques and serotonin on HSPs gene expression could provide valuable insights into the broader impacts of serotonin modulation on avian thermoregulation.

## Author Contributions

**Ladan Emadi**: conceptualization, methodology, investigation, supervision, project administration, writing–original draft, writing–review and editing. **Hamed Khasti**: methodology, investigation, writing–original draft, writing–review and editing. **Shahrzad Azizi**: methodology, investigation, writing–review and editing. **Elham Mohammadi**: methodology, writing–review and editing. **Hadi Tavakkoli**: methodology, writing–review and editing.

## Ethics Statement

All procedures were performed in compliance with the Guide for the Care and Use of Laboratory Animals by the National Academy of Sciences (National Institutes of Health Publication No. 86‐23) and after receiving institutional approval from the animal handling committee of the University of Kerman.

## Conflicts of Interest

The authors declare no conflicts of interest.

## Data Availability

The data that support the findings of this study are available on request from the corresponding author. The data are not publicly available due to privacy or ethical restrictions.
